# Estimation of Major Adverse Cardiovascular Events in Patients With Myocardial Infarction Undergoing Primary Percutaneous Coronary Intervention: A Risk Prediction Score Model From a Derivation and Validation Study

**DOI:** 10.3389/fcvm.2020.603621

**Published:** 2020-11-27

**Authors:** Xiaoxiao Zhao, Chen Liu, Peng Zhou, Zhaoxue Sheng, Jiannan Li, Jinying Zhou, Runzhen Chen, Ying Wang, Yi Chen, Li Song, Hanjun Zhao, Hongbing Yan

**Affiliations:** ^1^Fuwai Hospital, Chinese Academy of Medical Sciences and Peking Union Medical College, Beijing, China; ^2^Fuwai Hospital, Chinese Academy of Medical Sciences, Shenzhen, China

**Keywords:** myocardial infarction, primary percutaneous coronary intervention, MACE, derivation, validation

## Abstract

**Background:** The population with myocardial infarction (MI) undergoing primary percutaneous coronary intervention (PPCI) is growing, but validated models to guide their clinical management are lacking. This study aimed to develop and validate prognostic models to predict major adverse cardiovascular events (MACEs) in patients with MI undergoing PPCI.

**Methods and Results:** Models were developed in 4,151 patients with MI who underwent PPCI in Fuwai Hospital between January 2010 and June 2017, with a median follow-up of 698 days during which 544 MACEs occurred. The predictors included in the models were age, a history of diabetes mellitus, atrial fibrillation, chronic kidney disease, coronary artery bypass grafting, the Killip classification, ejection fraction at admission, the high-sensitivity C-reactive protein (hs-CRP) level, the estimated glomerular filtration rate, the d-dimer level, multivessel lesions, and the culprit vessel. The models had good calibration and discrimination in the derivation and internal validation with C-indexes of 0.74 and 0.60, respectively, for predicting MACEs. The new prediction model and Thrombolysis in Myocardial Infarction (TIMI) risk score model were compared using the receiver operating characteristic curve. The areas under the curve of the new prediction model and TIMI risk score model were 0.806 and 0.782, respectively (difference between areas = 0.024 < 0.05; *z* statistic, 1.718).

**Conclusion:** The new prediction model could be used in clinical practice to support risk stratification as recommended in clinical guidelines.

## What Is Already Known About This Topic?

Improving the quality and management of acute coronary syndrome contributed substantially to patients with cardiovascular disease. Although many clinical guidelines have been established, only a few tools are available to assess the incidence of major adverse cardiovascular events (MACEs) among patients with myocardial infarction (MI) undergoing primary percutaneous coronary intervention (PPCI) and to guide patients' clinician communication and long-term risk management.

## What Does This Article Add?

1. Calculated risk scores were used to develop a model that can further evaluate 1-, 2-, 3-, and 5-years risks of MACEs among patients with MI who underwent PPCI.
2. This risk score incorporates routine clinical data, biochemical tests, and coronary angiography findings, which are routinely evaluated in the clinical assessment of patients with MI who underwent PPCI. The risk score model can help doctors identify patients most at risk of MACEs.

## Introduction

Cardiovascular disease (CVD) has become the leading cause of mortality worldwide (1) and a major global economic burden (2). Early primary percutaneous coronary intervention (PPCI) has increased the survival rate and decreased the mortality rate, all-cause death rate, and incidence of recurrent myocardial infarction (MI) in patients with acute coronary syndrome (ACS) (3). Thus, the identification of pretreatment risk factors is beneficial to reduce the incidence of CVD in high-risk patients using multivariable prediction equations rather than single risk factors (4, 5). Clinical guidelines have provided direction for disease management; however, only a few tools can be used to assess the incidence of major adverse cardiovascular events (MACEs) among patients with MI undergoing PPCI and to guide patients' clinician communication and long-term risk management (6).

To achieve precision medicine, healthcare decisions, practices, and interventions should be individualized on the basis of each patient's predicted risk of disease. In this study, we sought to develop a risk score model to evaluate 1 -, 2 -, 3-, and 5-years risk for patients with MI who underwent PPCI. This article was prepared in accordance with the TRIPOD reporting checklist (Appendix File)^1^.

## Materials and Methods

### Study Design and Participants

A total of 4,151 consecutive patients with MI who underwent PPCI at Fuwai Hospital in Beijing, China, between January 2010 and June 2017 were enrolled. All patients were diagnosed with MI according to established guidelines (7, 8). The derivation cohort for this study comprised patients who had experienced MI at some time.

The enrolled patients provided informed consent, and the study was approved by the Ethics Committee of Fuwai Hospital. The study flowchart is shown in Appendix Figure 1.

### Definitions

Thrombolysis in Myocardial Infarction (TIMI) flow grade three levels less after PPCI was defined as a no-reflow phenomenon. For adverse events that occurred at follow-up, the following events were evaluated: all-cause mortality, cardiac mortality, MI recurrence, and stroke (ischemic stroke). The objective end-point index was evaluated using a single-blind method.

### Follow-Up Process

The patients were followed up at least 1 year after discharge. The health status of the enrolled patients was confirmed through telephone calls and review of health records, and this method was approved by the Review Board of Fuwai Hospital. The physicians in charge of the follow-up identified and extracted primary endpoints from hospital records, laboratory reports, and clinical notes in the event of death.

### Statistical Analyses

The normal distribution of the outcome variables was confirmed by the Kolmogorov–Smirnov test. For the randomization procedure, all enrolled patients were numbered 1 to 4,103. Then, cells were filled in with “=RAND ()” to create a list of randomized numbers and then sorted. The first 3,078 patients were derived queues, and the second 1,025 patients were validated queues. Baseline parameters during follow-up are presented as median [standard error (SE)] for continuous variables and as frequency and percentage for categorical variables in the table presenting the characteristics of the derivation cohort and validation cohort (Table 1). The variables included in the new prediction models were all pre-specified. Univariable Cox regression analysis (Appendix Table 2) was used to initially screen candidate factors with *P* < 0.2 for predicting MACE. The following variables were included to calculate major adverse CVD risk: sex, age, a history of hypertension, atrial fibrillation, a history of coronary artery bypass grafting (CABG), a history of PCI, diabetes status, blood pressure, the creatinine (Cr) level, the estimated glomerular filtration rate (eGFR), high-density lipoprotein cholesterol (HDL-C), low-density lipoprotein cholesterol (LDL-C), triglyceride (TG), lipase activator (LPA), and coronary angiography findings.

**Table 1:** The characteristics of derivation cohort and validation cohort.

**Variables**	**Derivation cohort**	**Validation cohort**	***P* value**
	***N* = 3,078**	***N* = 1,025**	
Age (years)	59.42 ± 0.217	58.82 ± 0.359	0.161
Male [% (*n*)]	78.91% (2,429)	77.37% (793)	0.158
Height (cm)	161.30 ± 0.04	161.7 ± 0.08	0.613
Weight (kg)	71.22 ± 0.29	70.86 ± 0.54	0.681
BMI (kg/m^2^)	25.91 ± 0.069	25.91 ± 0.116	0.997
Heart rate (beats per minute)	77 ± 0.28	78 ± 1.05	0.338
SBP (mm Hg)	124.45 ± 0.336	123.69 ± 0.581	0.254
DBP(mm Hg)	71.22 ± 0.291	70.86 ± 0.538	0.548
Hypertension [% (*n*)]	62% (1,903)	59% (604)	0.054
Diabetes [% (*n*)]	33% (1,012)	33% (335)	0.470
Hyperlipidemia [% (*n*)]	92% (2,831)	93% (957)	0.082
Smoking [% (*n*)]	59% (1,818)	59% (609)	0.307
Previous PCI [% (*n*)]	14% (419)	14% (146)	0.323
Previous CABG [% (*n*)]	1.2% (38)	1.0% (10)	0.316
Atrial fibrillation [% (*n*)]	6.2% (192)	6.0% (61)	0.403
CKD [% (*n*)]	7.8% (240)	8.6% (88)	0.229
**Laboratory examinations**			
HDL cholesterol at admission (mmol/L)	1.70 ± 0.02	1.97 ± 0.06	0.797
LDL cholesterol at admission (mmol/L)	2.75 ± 0.017	2.71 ± 0.028	0.307
Triglycerides at admission (mmol/L)	1.05 ± 0.005	1.05 ± 0.009	0.533
LPA at admission (mg/L)	263.87 ± 4.40	275.57 ± 8.00	0.200
hs-CRP at admission (mg/L)	7.59 ± 0.09	7.50 ± 0.16	0.622
d-Dimer at admission (μg/mL)	0.64 ± 0.03	0.68 ± 0.06	0.528
Peak level of d-dimer (μg/mL)	0.80 ± 0.04	0.80 ± 0.07	0.988
TnI at admission (ng/L)	3.58 ± 0.31	3.94 ± 0.63	0.573
Peak level of TnI (ng/L)	3.89 ± 0.25	3.98 ± 0.44	0.856
Crea at admission (μmol/L)	81.92 ± 0.45	82.73 ± 0.80	0.379
eGFR at admission (mL/min)	89.48 ± 1.49	89.96 ± 2.49	0.872
**Discharge medication regimen**			
Statin [% (*n*)]	91% (2,790)	91% (932)	0.287
Aspirin [% (*n*)]	96% (2,956)	96% (980)	0.412
Ticagrelor [% (*n*)]	74% (2,291)	74% (761)	0.508
ACEI/ARB [% (*n*)]	68% (2,094)	69% (709)	0.214
β-Blockers [% (*n*)]	85% (2,601)	85% (868)	0.379
Diuretic [% (*n*)]	28% (867)	28% (283)	0.401
Spironolactone [% (*n*)]	21% (649)	21% (216)	0.502
P2Y12 inhibitors	96% (2,958)	96% (986)	0.154
**Endpoint events**			
MACEs [% (*n*)]	11% (330)	12% (125)	0.107
Death [% (*n*)]	6.5% (200)	6.6% (68)	0.464
Recurrent MI [% (*n*)]	2.9% (90)	4.1% (42)	0.043
Stroke [% (*n*)]	1.7% (51)	2.0% (20)	0.307

*Continuous data are presented as mean ± SE (standard error); categorical variables are presented as % (n). BMI, body mass index; SBP, systolic blood pressure; DBP, diastolic blood pressure; PCI, percutaneous coronary intervention; CABG, coronary artery bypass grafting; CKD, chronic kidney disease; HDL-C, high-density lipoprotein cholesterol; LDL-C, low-density lipoprotein cholesterol; TnI, troponin I; TG, triglyceride; LPA, lipse activator; hs-CRP, high sensitive C-reactive protein; eGFR, estimated glomerular filtration rate; ACEI, angiotensin-converting enzyme inhibitor; ARB, angiotensin receptor blocker; MACE, major adverse cardiovascular events*.

The least absolute shrinkage and selection operator (LASSO) method was used to screen the independent variables to realize the reduction and simplification of the model and to prevent overfitting. Multivariable Cox regression was used to develop a novel prediction risk score for MACEs using all pre-specified variables (Appendix Table 1). In this study, time covered the period from the index assessment to the occurrence of the following events: death from other causes, CVD, MI recurrence, cerebrovascular disease, or end of follow-up. Missing data were handled by single imputation.

### LASSO Regression

At the beginning of the model establishment, all identified independent variables were selected to minimize model deviation caused by non-inclusion of important independent variables. Furthermore, the established model needs to find the set of independent variables with the strongest explanatory power for the dependent variables to improve the prediction accuracy. As a result, we included the LPA, HDL-C, and TG, which failed to have statistical significance by Cox regression in the LASSO regression. Therefore, index selection is significant in the modeling process. The 1996 LASSO algorithm is a compressed estimate method that simplifies the index set. A more refined model is obtained by constructing a function that compresses some coefficients and sets some coefficients to 0, 0.5, or minimization. LASSO regression is a biased estimation of data with complex collinearity and retains the advantage of contraction. LASSO programming is provided by the Lars algorithm software package of R language. Therefore, dimensionality reduction and variable selection can be achieved accurately by LASSO regression.

### Nomogram Prediction Model

The corresponding nomogram prediction model was drawn according to the regression coefficient of the selected independent variables. For the variables selected in the nomogram prediction model, values of the variables can correspond to the scores on the integral line at the top of the nomogram (the score ranged from 0 to 550 points) through the projection of the vertical line, and the total score can be obtained by adding the scores corresponding to the values of each variable. The cumulative occurrence probability of MACEs at 1, 2, 3, and 5 years can be obtained from the total score on the prediction line at the bottom of the nomogram. To reduce overfitting, the self-sampling method was used to verify the nomogram prediction model. Model discrimination was quantified using Harrell's c-statistic and calibration chart. Hypertension, hyperlipidemia, a history of PCI, smoking status, sex, blood pressure, body mass index, LDL-C, HDL-C, TG, left main (LM) artery lesion, and no-flow phenomenon were controlled to draw the receiver operating characteristic (ROC) curve. The LASSO method adopts the glmnet package of R language for variable selection and the RMS package of the R language for drawing and internal verification of the nomogram (c-index and calibration chart). Cox regression analysis was performed using the survival package. Stdca.r was used to draw the clinical decision curve. The main statistical analysis software used in this study was the R language version I 386 3.6.2. Other analyses were performed using SPSS Statistics version 20.0 (SPSS, Inc., Chicago, IL). All *P*-values were two-tailed, and statistical significance was determined at *P* < 0.05.

### Performance and Internal Validation of the New Risk Prediction Models

The 1-, 2-, 3-, and 5-years baseline survival probabilities of each model were obtained using R language version I 386 3.6.2 commands that were utilized to fit the models. Calibration performance was assessed graphically at 1-, 2-, 3-, and 5-years MACE risks by plotting the predicted 1-, 2-, 3-, and 5-years risks against the observed 1-, 2-, 3-, and 5-years risks. The flawlessly calibrated curve was represented by a diagonal line with a slope of 1. The observed 1-, 2-, 3-, and 5-years risks were obtained using the Kaplan–Meier method, and the slopes of regression lines comparing the predicted with the observed 1-, 2,- 3-, and 5-years risks were calculated. Standard statistical metrics of model and discrimination performance (*R*^2^, Harrell's c-statistic) were calculated. The calibration and discrimination performance of the equations developed in the derivation subcohort were assessed in the validation subcohort and compared with the performance of models developed in the entire cohort; baseline survival functions and hazard ratios (HRs) were also compared.

Indicators of internal verification included the c-index and calibration degree, which, respectively, represent the prediction accuracy and prediction consistency of the nomogram prediction model. The degree of calibration was represented by a calibration graph. ROC plotting was used for the survival ROC package. Owing to time constraints, experimental data from other research centers were not collected. Therefore, external validation was not performed, and this point is explained in the limitation section. The model was validated in a separate MI population, which was enrolled from July 2017 to December 2018. A total of 939 consecutive patients with MI who underwent PPCI at Fuwai Hospital in Beijing, China, were enrolled. However, the separate validation cohort underwent 1–2 years of follow-up; hence, the 1- and 2-years prediction models were validated. The performance and discrimination of a separate validation cohort were quantified using a calibration chart. The calibration graph indicated that the prediction model had good calibration and was shown in the Supplement.

### Comparison With Other Models

The accuracy of the new model and TIMI risk score model predicting MACEs among patients with MI who underwent PPCI was compared according to the area under the ROC (AUC) curve using a non-parametric test developed by DeLong et al. MedCalc for Windows version 18.2.1 (MedCalc Software, Mariakerke, Belgium) was used for comparison.

## Results

### Demographics of the Derivation Cohort and Validation Cohort

The study population included 4,151 men and women aged 24–97 years during risk assessment from January 1, 2010, to June 30, 2017 (Appendix Figure 1). Forty-eight people without follow-up data were excluded. Following randomized allocation, 3,078 people constituted the derivation cohort, and 1,025 (77% men) patients comprised the validation cohort. The median duration of follow-up was 698 days in the two cohorts. In the derivation cohort, the cumulative rate of the primary composite endpoint (MI, stroke, or all-cause death) was 11 during the follow-up period. Of these patients, 2.9% experienced recurrent MI, 1.7% experienced a stroke, and 6.5% died of any causes as their first event. In the validation cohort, there were 125 MACEs, of which 68 were all-cause deaths, 42 were MI recurrence, and 20 were cerebrovascular events. Participant characteristics are outlined in Table 1. Outcome events were obtained exclusively from follow-up databases between August 3, 2010, and March 11, 2019. The average age of the derivation cohort was 59.42 ± 0.217 years (mean ± SE), whereas the average age of the validation cohort was 58.82 ± 0.359 years (mean ± SE). No statistical differences were found between the two groups in terms of sex, heart rate, body mass index, blood pressure, disease history, laboratory examination, and discharge medication regimen. Therefore, they can be considered as two undifferentiated populations and can be used for model establishment and validation.

### Primary Screening by Univariate Cox Regression Analysis

The following variables are shown in Appendix Table 2: age (*P* = 0.053), hypertension (*P* = 0.013), diabetes mellitus (*P* < 0.0001), a history of atrial fibrillation (*P* < 0.0001), chronic kidney disease (CKD, *P* < 0.0001), a history of CABG (*P* < 0.0001), the Killip classification (*P* < 0.0001), ejection fraction (EF) grade (*P* < 0.0001), high-sensitivity C-reactive protein (hs-CRP) (*P* < 0.0001), eGFR (*P* < 0.0001), the d-dimer level (*P* < 0.0001), the Cr level (*P* < 0.0001), the use of intra-aortic balloon pump (*P* < 0.0001), a LM coronary artery lesion (*P* < 0.0001), the no-reflow phenomenon (*P* = 0.006), complete revascularization during hospitalization (*P* < 0.0001), triple-vessel lesions (*P* < 0.0001), culprit lesions including those in the left circumflex artery (LCX, *P* < 0.0001) and LM artery (*P* < 0.0001), the elevated LPA level (*P* = 0.343), the HDL-C level (*P* = 0.50), the TG level (*P* = 0.173), etc.

### Screening of the Independent Variables by the LASSO Method

Twenty-eight variables were filtered by the LASSO regression method, as shown in Appendix Figure 2. Thus, it is necessary to classify the variables by factorization and then use the as.matrix() function to convert the data from a non-matrix format to a matrix format before the R language “glmet” package can call the data. The filtering and cross-validation processes of the independent variables are shown in Appendix Figures 2A,B, respectively. Lambda.1se is the lambda value of the simplest model in the SE range, which identifies the model with excellent performance and the least number of independent variables. At this time, a total of 12 independent variables (age, a history of diabetes, a history of atrial fibrillation, a history of CABG, a history of CKD, the Killip classification, EF grade, an increase in hs-CRP level, a decrease in eGFR, an increase in d-dimer level, the culprit lesion, and mutivessel lesions) were included in the model.

### Establishment of a Multivariate Cox Regression Model and Risk Score Model

**Figure 1:**
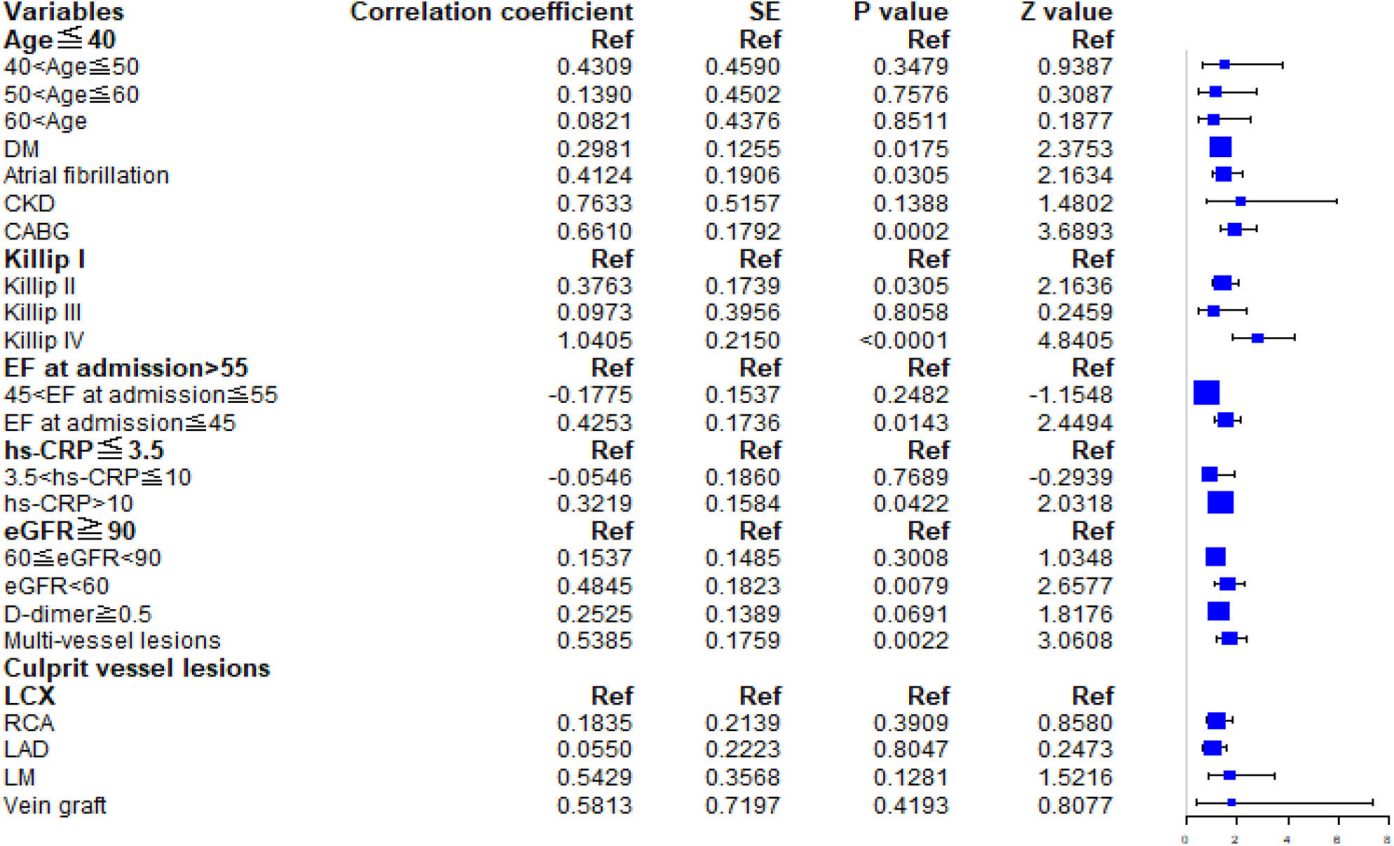
Forest plot. The HRs and 95% CIs for predictors in the multivariable Cox model for major adverse cardiovascular events. DM, diabetes mellitus; CKD, chronic kidney disease; CABG, coronary artery bypass grafting; EF, ejection fraction; hs-CRP, high-sensitivity C-reactive protein; eGFR, estimated glomerular filtration rate.

The multivariable Cox regression model established by the variables screened by LASSO method is shown in Appendix Table 1. Patients were categorized into four age groups: age ≤ 40 years, 40 < age ≤ 50 years, 50 < age ≤ 60 years, and age > 60 years. The group of patients aged 40–50 years [HR, 1.539; 95% confidence interval (CI), 0.626–3.783] was associated with a higher HR than other age groups for the incidence of MACE. In the multivariate Cox regression analysis, a history of diabetes mellitus (HR, 1.347; 95% CI, 1.054–1.723; *P* = 0.0175), atrial fibrillation (HR, 1.511; 95% CI, 1.040–2.195; *P* = 0.0305), CABG (HR, 1.937; 95% CI, 1.363–2.752; *P* = 0.0002), EF grade at admission ≤45 (HR, 1.530; 95% CI, 1.089, 2.150; *P* = 0.0143), and multivessel lesions (HR, 1.713; 95% CI, 1.214, 2.419; *P* = 0.0022) were relevant factors for MACEs during follow-up. The forest plot is shown in Figure 1.

### Nomogram Depiction and Evaluation of the Risk Prediction Model

The model and discrimination metrics indicated that the risk equations performed better in predicting MACE. Additional variables available in the prediction model, when added to the LASSO regression models, are shown in Appendix Table 1. Age, diabetes mellitus, a family history of CKD, atrial fibrillation, CABG, the Killip score, EF grade at admission, hs-CRP, eGFR, d-dimer, the number of culprit lesions, and multivessel lesions were all statistically significant predictors of MACE risk (Appendix Table 1).

### Interpretation of the Newly Established Risk Score

**Figure 2:**
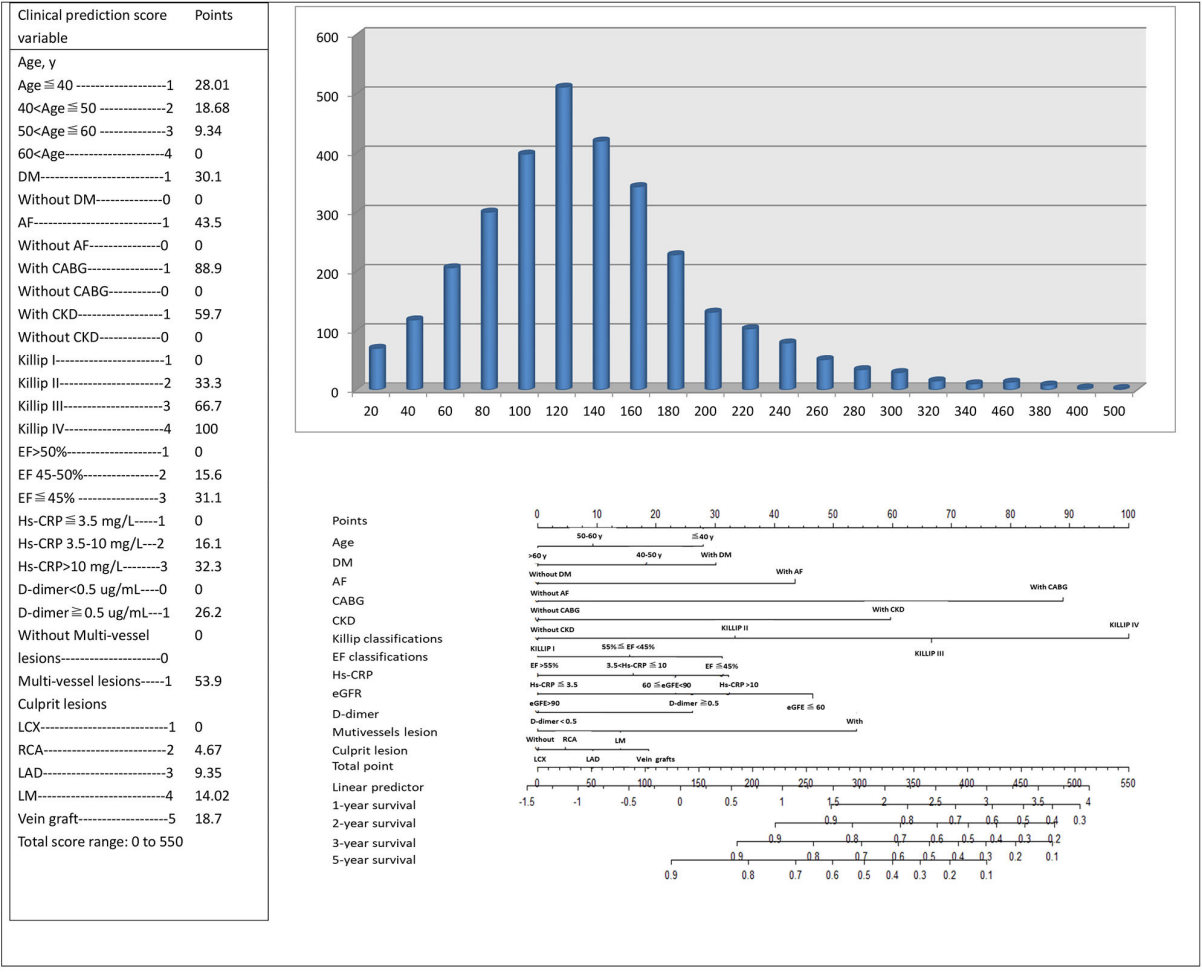
The risk score nomogram for bedside application. Histogram refers to the score distribution in the derivation cohort. For the variables selected in the nomogram model, the values of different variables can correspond to different scores on the integral line at the top of the nomogram (the score range is 0–550 points) through the projection of the vertical line, and the total score can be obtained by adding up the scores corresponding to the values of each variable. The cumulative occurrence probability of MACEs in 3 and 5 years can be obtained from the total score on the prediction line at the bottom of the nomogram.

Scoring was performed during hospitalization to predict long-term events by physicians. For the variables selected in the nomogram prediction model, the values of variables can correspond to the scores on the integral line at the top of the nomogram (the score ranged from 0 to 550 points) through the projection of the vertical line, and the total score can be obtained by adding the scores corresponding to the values of each variable. The cumulative occurrence probability of MACEs at 1, 2, 3, and 5 years can be obtained from the total score on the prediction line at the bottom of the nomogram. The scores, ranging from 0 to 550 points, were assigned as follows: age < 40 years, 28.01; age 40–50 years, 18.68; age 50–60 years, 9.34; age ≥60 years, 0; diabetes mellitus, 30.1; without diabetes mellitus, 0; atrial fibrillation, 43.5; without atrial fibrillation, 0; history of CABG, 88.9; without history of CABG, 0; history of CKD, 59.7; without history of CKD, 0; Killip I, 0; Killip II, 33.3; Killip III, 66.7; Killip IV, 100; EF at admission ≤45%, 31.1; EF at admission between 45% and 55%, 15.6; EF at admission >55%, 0; hs-CRP concentration that varies from 3.5 to 10 mg/L and over 10 mg/L, 16.1 and 32.3, respectively; hs-CRP concentration ≤3.5 mg/L, 0; eGFR of 60–90 mL/min and <60 mL/min, 23.3 and 46.5, respectively; eGFR >90 mL/min, 0; d-dimer concentration ≥ 0.5 μg/mL, 26.2; d-dimer concentration <0.5 μg/mL, 0; mutivessel lesions, 53.9; without multivessel lesions, 0; vein graft culprit lesion, 18.7; LM culprit lesion, 14.02; LAD culprit lesion, 9.35; right coronary artery culprit lesion, 4.67; and LCX culprit lesion, 0. The distribution of the risk score is shown in Figure 2. With the increase in the total score of the nomogram prediction model, the corresponding 1-, 2-, 3-, and 5-years risk of MACEs increased (Figure 2). Despite the evaluation of the reliability and validity of the C-index, it provides a reliable tool for evaluating the model. The C-index was 0.74 in the derivation cohort and 0.60 in the validation cohort. Appendix Figure 3 shows the ROC curves for the discriminatory value of the 3- and 5-years evaluation performance of the risk prediction model. Appendix Figure 3 shows the survival ROC curves for evaluating the performance of the new risk prediction model at 1 year (A, AUC = 0.715, cutoff point = 15.29396), 2 years (B, AUC = 0.692, cutoff point = 15.08561), 3 years (C, AUC = 0.674, cutoff point = 15.04044), and 5 years (D, AUC = 0.638, cutoff point = 14.87151) in the derivation cohort.

**Figure 3:**
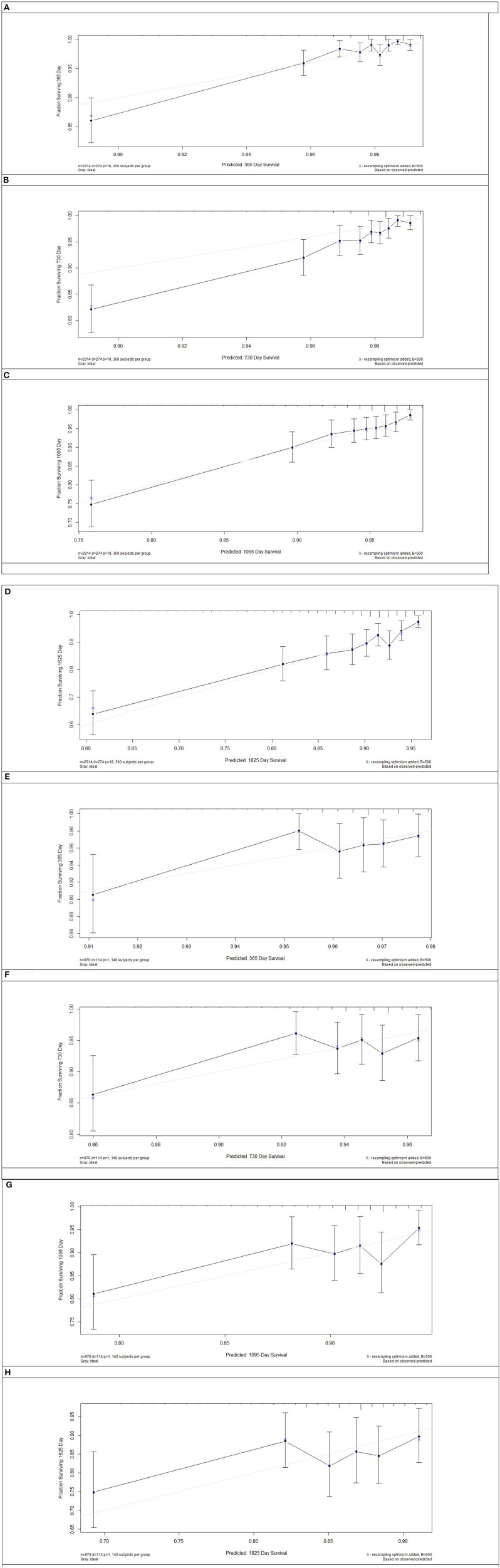
Risk score calibration in the derivation cohort and internal validation cohort. The major adverse cardiovascular events risk score of 1 **(A)**, 2 **(B)**, 3 **(C)**, and 5 years **(D)** in the derivation cohort and 1 **(E)**, 2 **(F)**, 3 **(G)**, and 5 years **(H)** in the validation cohort. Calibration is shown as the estimated risk against survival from Kaplan–Meier analysis. Gray line = perfect calibration.

**Figure 4:**
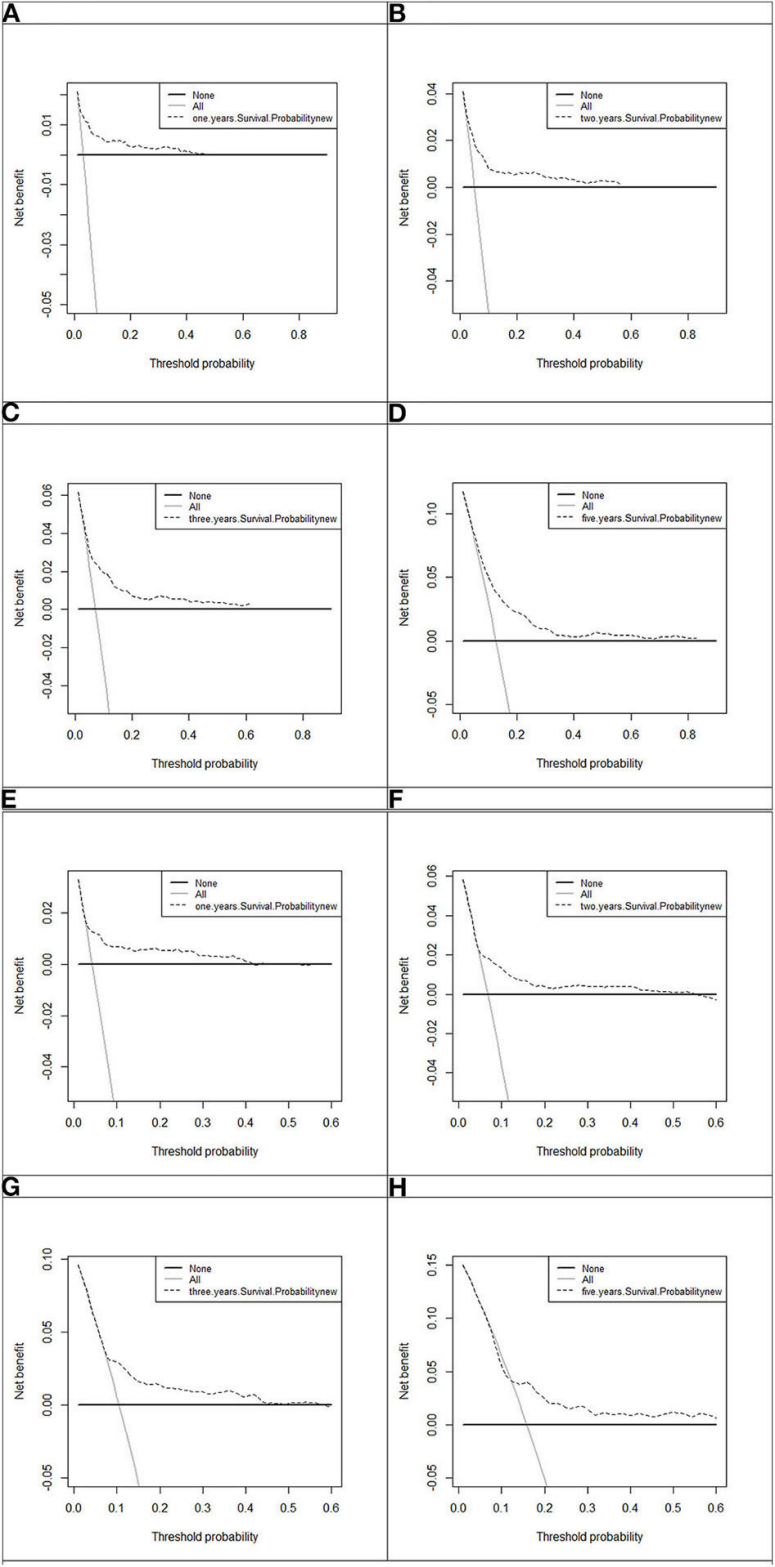
Decision curve analysis of 1, 2, 3, and 5 years in the derivation and 1, 2, 3, and 5 years in the validation cohort. The black line, assume no patients without disease (represented by None, i.e., the horizontal line). Gray line, all the MACE patients (represented by All, i.e., the oblique line). Dotted line represents the new established model. **(A)** One-year decision curve analysis in the derivation cohort; **(B)** 2-years decision curve analysis in the derivation cohort; **(C)** 3-years decision curve analysis in the derivation cohort; **(D)** 5-years decision curve analysis in the derivation cohort; **(E)** 1-year decision curve analysis in the validation cohort; **(F)** 2-years decision curve analysis in the validation cohort; **(G)** 3-years decision curve analysis in the validation cohort; **(H)** 5-years decision curve analysis in the validation cohort.

**Figure 5:**
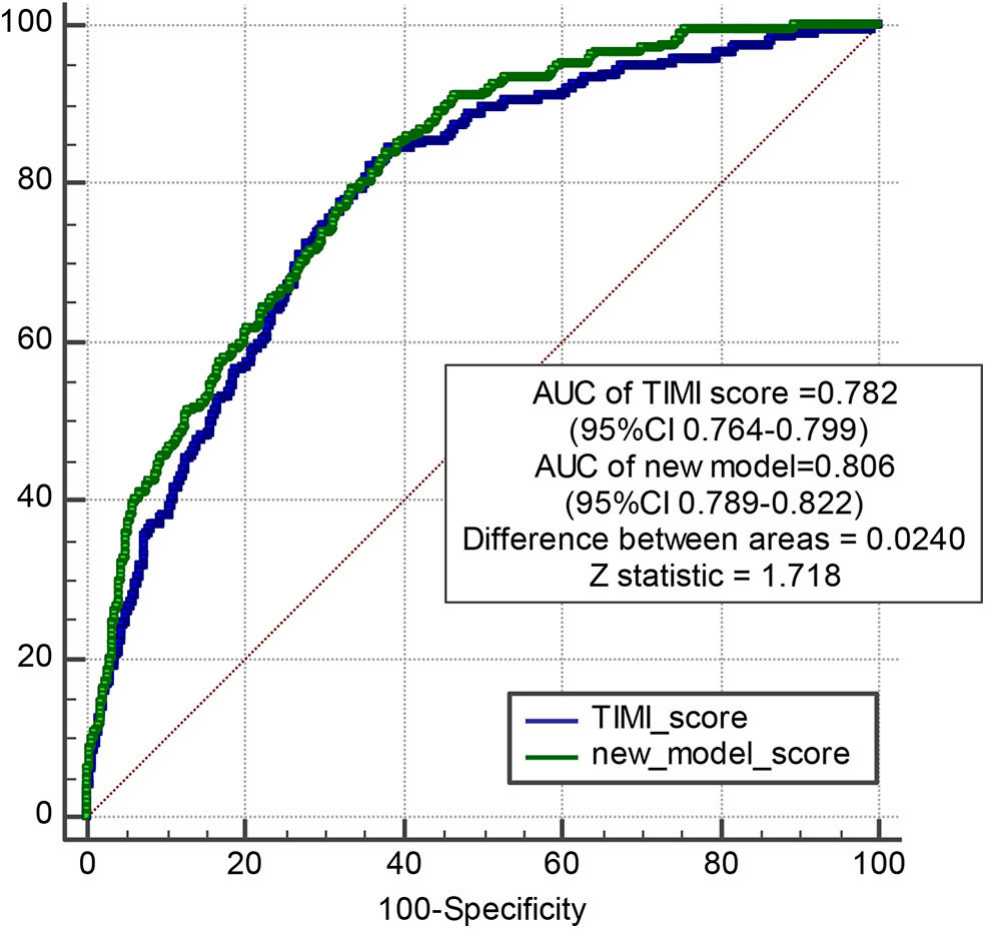
Pairwise comparison of ROC curves between new model and TIMI risk score model. The AUC of new model is 0.806, and the AUC of TIMI score is 0.782. Difference between areas = 0.0240 < 0.05; *z* statistic, 1.718.

Model discrimination was quantified using Harrell's c-statistic and calibration chart. The predicted vs. observed 1-, 2-, 3-, and 5-years risk plots for MACEs using the risk prediction model showed excellent calibration performance (Figures 3A–H). Figure 3 shows the MACE risk scores at 1, 2, 3, and 5 years in the derivation cohort (Figures 3A–D) and validation cohort (Figures 3E–H). Calibration is indicated by the estimated risk against survival from the Kaplan–Meier analysis. The gray line indicates perfect calibration. Figures 4A–H show the decision curve analysis of 1, 2, 3, and 5 years in the derivation and validation cohorts. Assuming that we choose to predict the 12% risk of MACEs and treatment, modeling queue Decision Curve Analysis (DCA) curves showed that if the new prediction model is used in every 10,000 people at the first year of follow-up, 50 people can benefit from this model without influencing any other person's interests, with 100 in 10,000 people at the second year, 200 in 10,000 people at the third year, and 500 in every 10,000 people at the fifth year. The internal validation queue DCA curves show that if the new prediction model is used in 10,000 people during the first year of follow-up, 100 people can benefit from the model without influencing any other person's interests, with 180 in 10,000 people at the second year, 250 in 10,000 people at the third year, and 500 from every 10,000 at the fifth year. Figure 5 compares the predictive efficiency between the new prediction model and the TIMI risk score model. The AUC of the new prediction model was 0.806, and the AUC of the TIMI risk score model was 0.782 (difference between areas = 0.024; *z* statistic, 1.718). Appendix Figure 4 shows the calibration graph of the cohorts and the excellent calibration performance.

## Discussion

Patient management and assessment should be individualized and precise to ensure the sustainable development of the contemporary healthcare system. For patients with ACS, care should be appropriate for the disease type and stage; however, only a few tools can be used to assist medium- to long-term management of patients with MI undergoing PPCI (9). Patients with MI do not have the same risk of recurrence, and the risk level is still relevant even after the 6-months period as predicted by the most accurate score (10, 11).

We have developed a risk score model to evaluate the 3- and 5-years risk probability for patients with MI who underwent PPCI, and this model can be used by specialists and primary healthcare professionals to enhance risk management and assessment. This risk score model incorporates routine clinical data on serum inflammatory factors and coronary angiography findings by integrating time since event, and it allows re-evaluation of the risk of MACEs 3 years or more following PPCI. The established risk score may be used to inform decisions about novel therapies and be tested in the context of changes in quantifiable risk.

The enrolled patients with acute MI who underwent PPCI are the most appropriate population to develop or validate a risk prediction model for MACE. However, similar cohorts are rare. While there was a statistically significant difference between the derivation and validation cohorts in terms of recurrent MI (*P* = 0.043), the model was established by derivation only, and the models had good calibration and discrimination in derivation and internal validation by the C-index and calibration graph for predicting MACE. Therefore, the proposed model does not increase the risk of recurrent MI.

### How This Risk Scoring System Can and Should Influence Patient Treatment

Clinical risk factors and biochemical measurements of serum and coronary angiography findings, which are easily obtained and routinely collected at admission, are incorporated into our nomogram prediction model, which takes advantage of a novel screening method and presents as a robust predictive model of MACE. The nomogram incorporating coronary angiography results can be used to inform patients about their future risk up to 3 and 5 years and be a useful tool for clinical practice. Furthermore, the results may be used as reference for preventive therapy, such as improving renal function, enhancing heart function, and lowering inflammation in patients with a high risk of MACE.

We performed a comparative study between the new prediction model and TIMI risk score model to evaluate the effect of preventive therapeutic strategies. The results show that the new prediction model has better effects and is more suited for patients with MI undergoing PPCI. MACEs could be considered an appropriate endpoint free from misclassification bias. Furthermore, we identified age, especially age <40 years, as a risk factor for MACEs following PPCI. In this study, patients were categorized into four age groups (age ≤40 years, 40 < age ≤50 years, 50 < age ≤60 years, age >60 years), which were assigned 28.01, 18.68, 9.34, and 0 points in the nomogram. These results were consistent with those of Dawson et al. (12); that is, the incidence rate ratio of patients aged 35–39 years (i.e., 28.1) was higher than those aged >85 years (i.e., 0.65).

### Contribution of the Prediction Model

Robust evidence highlights the tremendous contribution of inflammation to the development of plaque, vulnerability, and progression of ACS. Higher plasma concentrations of inflammatory mediators such as CRP and d-dimer were significantly correlated with a greater risk for MACE. The early inflammatory response is generated by proinflammatory cytokines, with important biological functions in the cascading inflammatory reaction and critical role in the occurrence and development of acute ischemic injury. T lymphocytes, mast cells, and macrophages play critical roles in the pathogenesis of MI treated with PPCI. Furthermore, serum biomarkers reflecting systemic inflammatory levels may help establish proper clinical management and therapeutic schedules. In this study, the serum concentration of hs-CRP that varied from 3.5 to 10, and >10 was assigned 15.9 and 31.7 points, respectively. The scores of the high d-dimer concentration (≥0.5) and triple-vessel lesions were 27.2 and 34.2, respectively. Inflammation markers including hs-CRP and d-dimer contributed substantially to the prediction score model after screening by LASSO regression. Moreover, the role of inflammation level has been proved in plaque ruptures. Therefore, anti-inflammatory approaches could benefit patients by significantly reducing levels of serum inflammation biomarkers. Acute MI can be viewed on a life-course continuum, progressing from the presence of risk factors to occurrence of subclinical atherosclerosis and MI induced by plaque rupture. Numerous risk scores contribute to the management and primary prevention of MI (13–15). However, few equivalent scores are available for use in patients with acute MI undergoing PPCI. For these patients, cardiac imaging, coronary angiography, and advanced biomarkers are routinely available at the time of admission, so it is convenient to include them in a scoring system for this setting for long-term management.

### Benefits of the Prediction Scoring Model

In the past few decades, the incidence of CVD worldwide has decreased significantly as a consequence of preventive treatments (16, 17). Most published equations and models of CVD, including the American Heart Association PCEs/2013 American College of Cardiology, are likely out of date (18). The predictive performance of the new model to identify 3- and 5-years MACE risk was calibrated. We found that adding routinely available measures of coronary angiography, renal function indices, and other easily measured predictors identified groups of patients whose risk would otherwise be appreciably underestimated or overestimated. We developed equations predicting 5-years risk rather than the more common 10-years risk because most trials of CVD risk reduction have ~5 years of follow-up. Potential predictors and MACE definitions were prespecified to reduce overfitting. To assess the degree of overoptimism of re-substitution validation, sensitivity analyses were performed by dividing the cohort into derivation and validation subcohorts. We replicated the equation development and model performance procedures (Appendix) in the validation subcohorts to evaluate the performance. Equation coefficients, baseline survival functions, and performance metrics were similar irrespective of whether the whole cohort or derivation cohort was used to develop the risk score model.

Both the time of risk assessment post-event and CVD type are main factors used to evaluate the performance of the risk score for secondary prevention. The CALIBER group (19) enrolled 102,023 stable coronary artery disease patients with a mean follow-up of 4.4 years and developed a risk score for this population. They used the new model to identify high-risk patients (defined by guidelines as 3% annual mortality) and support management decisions. A recent study randomized patients post-ACS to receive clopidogrel (control) or ticagrelor on the basis of their ischemic and bleeding risks predicted by the GRACE and CRUSADE scores, suggesting that the use of an appropriate risk scoring system to guide antiplatelet therapy after ACS is safe and can improve clinical outcomes (20). Many standard clinical recommendations have been applied to all patients post-ACS by evaluating the risk factor modification and medications. Such programs have been successfully established in the context of the primary prevention and understanding of the life course of CVD. In a recent systematic review (21), 10,363 models were identified, and most CVD risk prediction models were developed in Europe and North America. The study recommended the use of uniform definitions, preferably *International Classification of Diseases*–coded events and outcome definitions. While the variables measured by coronary angiography are independently associated with MACE, most published equations include only a limited number of predictors (typically age, sex, smoking, diabetes, blood pressure, and blood lipids). One of the most comprehensive equations was from the UK QRISK3 (13), which included 22 variables, but it was difficult to assess and use outside the United Kingdom. Separate equations have been developed in the United States for black and white people, but Asians are not represented (15).

Subsequent management and risk prediction titrated against risk are all required to improve clinical outcomes for patients with MI undergoing PCI. The risk scores presented herein can be implemented alongside further medical investigations to support therapeutic decision-making and guide clinicians and patients toward individualized healthcare. A lower risk score in the post-PPCI setting would not be the reason to withdraw medications. Rather, it is a tool to enhance clinician–patient interactions to reinforce risk factor modification. The advantages of the risk tools are likely dependent on the local healthcare environment and healthcare settings. Thus, it is a logical evolution to use this experience in patients with MI undergoing PPCI.

### Post-procedural Inflammation Level and Renal Function Are Associated With Increased Risk of MACE

Incorporating acute phase inflammation factors into the prediction model of long-term events based on previous literature is reasonable. Robust evidence (22–24) has shown that levels of inflammation markers, including hs-CRP and d-dimer, are constantly associated with worse mortality among patients with ACS who underwent PCI. Our previous study (25) revealed that during a median follow-up of 727 days, both low and high post-procedural hs-CRP levels were associated with a higher risk of death in patients with ACS who underwent PCI. Hs-CRP is the key marker of the interleukin (IL)-1β/IL-6/CRP pathway to synthesize and recruit leukocyte after myocardial damage (26). Hs-CRP and d-dimer could indirectly regulate the infiltration of neutrophils and macrophages into the infarcted myocardium and could lead to the delayed cleaning of apoptotic or necrotic cardiomyocytes, increasing myocardial fibrosis, and reducing EF, resulting in worse clinical outcomes during long-term follow-up. In this study, high levels of inflammation markers (d-dimer >0.5 mg/L and hs-CRP >10 mg/L) were risk factors of MACEs during a median follow-up of 698 days, which is consistent with previous studies.

CKD is correlated with a high risk of mortality from CVD (27), and patients with CVD-induced CKD are more likely to have a worse outcome during follow-up (28). Patients with severe CKD are at a high risk of diffuse obstructive coronary atherosclerosis (29). The absence of reliable risk estimates in patients with CKD limits the ability of clinicians to make evidence-based decisions. A previous study (30) reported that eGFR levels in the range of 45 to 60 mL/min/1.73 m^2^ and <45 mL/min/1.73 m^2^ predicted MACEs (adjusted HRs, 1.25 and 2.26, respectively). In the present study, we included CKD history and eGFR levels on admission into the prediction model. eGFR tested post-PPCI reflects the renal function at that time and is non-repeatable, given the variations in CKD history.

### No-Flow Phenomenon Is Associated With Increased Risk of MACE

A substantial proportion of patients still had myocardial tissue hypoperfusion after PPCI, which is termed as no-reflow phenomenon caused by microvascular obstruction (31–33). Previous studies have reported an association between the no-flow phenomenon and adverse clinical outcomes after ST-elevation MI (34–38). A metaregression study (39) reported that the no-flow phenomenon was independently correlated with increased 1-year all-cause mortality and 1-year heart failure hospitalization in the fully adjusted model. Although no-reflow phenomenon occurs in the acute phase of ACS, it is relatively significant in predicting events after more than 1 year of follow-up.

## Strengths and Limitations

This study developed a risk score model to evaluate 3- and 5-years risks of patients with MI who underwent PPCI. The researchers followed strict inclusion and exclusion criteria, which enabled a reasonably streamlined and comparable hospital flow for all patients. The model incorporated variables including routine clinical data, serum inflammatory factors, coronary angiography findings, and other relevant clinical parameters that are commonly included in clinical assessment. These variables are routinely documented in electronic health records; therefore, their collection is not linked with extra costs.

Nevertheless, this study has several potential limitations. First, it is a single-center study of an ethnic population that is not diverse. Second, the large size of the dataset used to develop the models reduces the likelihood of overfitting. Third, d-dimer, hs-CRP, and EF are not routinely obtained as the standard of care for MI patients. Inclusion of these variables may increase the chance of having missing information and inability to calculate the risk score. Finally, patients have been enrolled over a long time period, which could have confounding effects due to improvements in interventional techniques and progress in medication.

## Conclusion

In summary, we present risk prediction models for estimating the risk for MACEs on the basis of clinical parameters that are commonly available in all patients with MI undergoing PPCI. These models can be implemented alongside further medical investigations to support therapeutic decision-making. However, as with any new risk prediction model, further independent evaluation is required in different settings, including different geographic locations and healthcare organizations, to guide application in clinical management and practice.

## Data Availability Statement

The data analyzed in this study is subject to the following licenses/restrictions: The datasets used and/or analyzed during this study are available from the corresponding author on reasonable request. Requests to access these datasets should be directed to hbyanfuwai2018@163.com.

## Author Contributions

XZ and HY: conception and design. HY: administrative support. HY, CL, and PZ: provision of study materials or patients. XZ, CL, PZ, ZS, JL, JZ, RC, and YW: collection and assembly of data and data analysis and interpretation. All authors: manuscript writing and final approval of manuscript.

## Conflict of Interest

The authors declare that the research was conducted in the absence of any commercial or financial relationships that could be construed as a potential conflict of interest.

## Footnotes

^1^The authors have completed the TRIPOD reporting checklist.
