# Supplementary material for: Estimation of Major Adverse Cardiovascular Events in Patients With Myocardial Infarction Undergoing Primary Percutaneous Coronary Intervention: A Risk Prediction Score Model From a Derivation and Validation Study
Source: Front Cardiovasc Med. 2020 Nov 27;7:603621. doi: 10.3389/fcvm.2020.603621 (PMC7728669; doi:10.3389/fcvm.2020.603621)

**Online Appendix for the following Article**

**TITLE:** Estimation of MACE among MI patients undergoing primary PCI: a risk prediction score project from derivation and validation study

**AUTHORS:** Xiaoxiao Zhao MD, Chen Liu MD, Peng Zhou MD, Zhaoxue Sheng MD, Jiannan Li MD, Jinying Zhou MD, Runzhen Chen MS, Ying Wang MD, Yi Chen MD, Li Song, Hanjun Zhao MD, Hongbing Yan MD PHD

# APPENDIX

This appendix has been provided by the authors to give readers additional information about their work.

| **Contents** |
| --- |
| Supplementary Materials |
| I Appendix table1 Results of Mutivariate Cox proportional hazards model applied to assess correlates of MACE |
| II Appendix table2 Results of Univariate Cox proportional hazards model applied to assess correlates of MACE |
| III Appendix 3 the processes and methods of modeling |
| IV Appendix Figure1 Flow chart of study enrollment. |
| V Appendix Figure 2 LASSO regression |
| VI Appendix figure 3 Survival ROC curve of 3 -year and 5-year cumulative MACE incidence predictive risk in the derivation cohort |
| VII Appendix figure4 Calibration graph in the separate cohort |

**Supplementary Materials**

**Appendix Figure 1:** Flow chart of study enrollment. PCI, percutaneous coronary intervention

**Appendix Figure 2:** LASSO regression

**Appendix Figure 3:** survival ROC curve

Survival receiver operating characteristic curves for evaluating the performance of the new risk predicted model of 3-year (A, AUC=0.733, cutoff point=3.63178) and 5-year (B, AUC=0.714, cutoff point=2.78009) in the derivation cohort. The areas under ROC curve are shown for the graph. AUC, area under the curve; ROC, survival receiver operating characteristic; TP, true positive; FP, false positive.

**Appendix Figure 4:** ROC curve of 3-year and 5-year cumulative MACE incidence predictive risk in the derivation and validation cohort. A, ROC curve of 3-year cumulative MACE incidence predictive risk in the derivation cohort (AUC=0.725, p<0.0001, CI 95% [0.678, 0.773]); B, ROC curve of 3-year cumulative MACE incidence predictive risk in the validation cohort (AUC=0.822, p<0.0001, CI 95% [0.754, 0.890]). C, ROC curve of 5-year cumulative MACE incidence predictive risk in the derivation cohort (AUC=0.739, p<0.0001, CI 95% [0.670, 0.809]).D, ROC curve of 5-year cumulative MACE incidence predictive risk in the validation cohort (AUC=0.872, p<0.0001, CI 95% [0.798, 0.945]).

Appendix figure 5 Calibration graph in the separate cohort

Appendix table 1: Results of Mutivariate Cox proportional hazards model applied to assess correlates of MACE

Appendix table 2: The processes and methods of modeling

Appendix table 1: Results of Mutivariate Cox proportional hazards model applied to assess correlates of MACE

| Variables | Correlation coefficient | SE | P value | Z value | HR (95%CI) |
| --- | --- | --- | --- | --- | --- |
| Age≦40 | Ref | Ref | Ref | Ref | 1 |
| 40<Age≦50 | 0.4309 | 0.4590 | 0.3479 | 0.9387 | 1.539(0.626,3.783) |
| 50<Age≦60 | 0.1390 | 0.4502 | 0.7576 | 0.3087 | 1.149(0.476,2.777) |
| 60<Age | 0.0821 | 0.4376 | 0.8511 | 0.1877 | 1.086(0.460,2.560) |
| DM | 0.2981 | 0.1255 | 0.0175 | 2.3753 | 1.347(1.054,1.723) |
| [Atrial fibrillation](http://www.baidu.com/link?url=mme3aA3sg8G0gGf26Lb5Gh3kriqhyTVcYFOh8BiFsvaO11zuD7IrtT8QZ0KufseQRj--2ZGStOFSTKRGHHXgij21GeOXq26qyiBliqxPs7tLCrUR9BXd5hkDAUovAoo3) | 0.4124 | 0.1906 | 0.0305 | 2.1634 | 1.511(1.040,2.195) |
| CKD | 0.7633 | 0.5157 | 0.1388 | 1.4802 | 2.145(0.781,5.894) |
| CABG | 0.6610 | 0.1792 | 0.0002 | 3.6893 | 1.937(1.363,2.752) |
| Killip I | Ref | Ref | Ref | Ref | 1 |
| Killip II | 0.3763 | 0.1739 | 0.0305 | 2.1636 | 1.457(1.036,2.049) |
| Killip III | 0.0973 | 0.3956 | 0.8058 | 0.2459 | 1.102(0.508,2.394) |
| Killip IV | 1.0405 | 0.2150 | <0.0001 | 4.8405 | 2.831(1.858,4.314) |
| EF at admission>55 | Ref | Ref | Ref | Ref | 1 |
| 45<EF at admission≦55 | -0.1775 | 0.1537 | 0.2482 | -1.1548 | 0.837(0.610,1.132) |
| EF at admission≦45 | 0.4253 | 0.1736 | 0.0143 | 2.4494 | 1.530(1.089,2.150) |
| hs-CRP≦3.5 | Ref | Ref | Ref | Ref | 1 |
| 3.5<hs-CRP≦10 | -0.0546 | 0.1860 | 0.7689 | -0.2939 | 0.947(0.657,1.882) |
| hs-CRP>10 | 0.3219 | 0.1584 | 0.0422 | 2.0318 | 1.380(1.011,1.560) |
| eGFR≧90 | Ref | Ref | Ref | Ref | 1 |
| 60≦eGFR<90 | 0.1537 | 0.1485 | 0.3008 | 1.0348 | 1.166(0.872,1.560) |
| eGFR<60 | 0.4845 | 0.1823 | 0.0079 | 2.6577 | 1.623(1.136,2.320) |
| D-dimer≧0.5 | 0.2525 | 0.1389 | 0.0691 | 1.8176 | 1.287(0.980,1.690) |
| Multi-vessel lesions | 0.5385 | 0.1759 | 0.0022 | 3.0608 | 1.713(1.214,2.419) |
| Culprit vessel lesions |  |  |  |  |  |
| LCX | Ref | Ref | Ref | Ref | 1 |
| RCA | 0.1835 | 0.2139 | 0.3909 | 0.8580 | 1.201(0.790,1.827) |
| LAD | 0.0550 | 0.2223 | 0.8047 | 0.2473 | 1.057(0.683,1.634) |
| LM | 0.5429 | 0.3568 | 0.1281 | 1.5216 | 1.721(0.855,3.464) |
| Vein graft | 0.5813 | 0.7197 | 0.4193 | 0.8077 | 1.788(0.436,7.329) |

B, correlation coefficient; SE, standard error; HR, hazard ratio; ref, reference; DM, diabetes mellitus; EF, ejection fraction; CKD, chronic kidney disease; eGFR, estimated glomerular filtration rate; CABG, coronary artery bypass grafting; hs-CRP, high sensitive C-reactive protein; MACE, major adverse cardiovascular events

Appendix table 2: Results of Univariate Cox proportional hazards model applied to assess correlates of MACE

| Variables | B | Wald | HR | CI (95%) | P值 |
| --- | --- | --- | --- | --- | --- |
| Age≦40 | Ref | 10.935 | Ref | Ref | 0.053 |
| 40<Age≦50 | 0.143 | 0.129 | 1.154 | 0.529-2.517 | 0.720 |
| 50<Age≦60 | -0.272 | 0.484 | 0.761 | 0.353-1.641 | 0.487 |
| 60<Age≦70 | 0.063 | 0.028 | 1.065 | 0.509-2.230 | 0.867 |
| 70<Age≦80 | -0.206 | 0.285 | 0.814 | 0.382-2.230 | 0.593 |
| Age>80 | -0.695 | 2.262 | 0.499 | 0.202-1.234 | 0.133 |
| Male | 0.015 | 0.013 | 1.015 | 0.792-1.300 | 0.908 |
| Optimal BP | Ref | 8.166 | Ref | Ref | 0.043 |
| Normal BP | -0.107 | 0.413 | 0.899 | 0.649-1.244 | 0.521 |
| Hi Normal BP | 0.102 | 0.329 | 1.107 | 0.783-1.566 | 0.566 |
| hypertension | 0.340 | 5.220 | 1.405 | 1.050-1.881 | 0.022 |
| Killip I | Ref | 180.842 | Ref | Ref | 0.000 |
| Killip II | 0.618 | 14.102 | 1.855 | 1.344-2.562 | 0.000 |
| Killip III | 0.783 | 4.720 | 2.188 | 1.080-4.434 | 0.003 |
| Killip IV | 2.167 | 178.005 | 8.736 | 6.354-12.011 | 0.000 |
| IABP using | 0.649 | 17.897 | 1.914 | 1.417-2.586 | 0.000 |
| EF at admission>55 | Ref | 39.811 | Ref | Ref | 0.000 |
| 45<EF at admission≦55 | -0.047 | 0.109 | 0.954 | 0.722-1.261 | 0.742 |
| EF at admission≦45 | 0.782 | 29.493 | 2.185 | 1.648-2.897 | 0.000 |
| DTB time | 0.059 | 0.157 | 1.061 | 0.792-1.421 | 0.691 |
| Risk factors |  |  |  |  |  |
| Hypertension | 0.305 | 6.238 | 1.357 | 1.068-1.724 | 0.013 |
| Diabetes | 0.482 | 17.289 | 1.619 | 1.290-2.032 | 0.000 |
| [Atrial fibrillation](http://www.baidu.com/link?url=mme3aA3sg8G0gGf26Lb5Gh3kriqhyTVcYFOh8BiFsvaO11zuD7IrtT8QZ0KufseQRj--2ZGStOFSTKRGHHXgij21GeOXq26qyiBliqxPs7tLCrUR9BXd5hkDAUovAoo3) | 0.921 | 29.483 | 2.512 | 1.802-3.503 | 0.000 |
| CKD | 1.217 | 66.478 | 3.376 | 2.520-4.523 | 0.000 |
| Previous CABG | 1.260 | 15.308 | 3.527 | 1.876-6.630 | 0.000 |
| Previous PCI | 0.275 | 2.712 | 1.317 | 0.949-1.827 | 0.100 |
| Inflammatory factors |  |  |  |  |  |
| D-dimer≧0.5 mmol/L | 0.667 | 32.021 | 1.949 | 1.547-2.456 | 0.000 |
| hs-CRP≦3.5 mmol/L | Ref | 23.296 | Ref | Ref | 0.000 |
| 3.5<hs-CRP≦10 mmol/L | 0.072 | 0.169 | 1.075 | 0.762-1.516 | 0.681 |
| hs-CRP>10 mmol/L | 0.593 | 16.462 | 1.809 | 1.359-2.409 | 0.000 |
| Renal function |  |  |  |  |  |
| Crea<44 mmol/d | Ref | 41.299 | Ref | Ref | 0.000 |
| 44≦Crea≦133 mmol/d | -0.167 | 0.158 | 0.846 | 0.371-1930 | 0.691 |
| Crea>133 mmol/d | 1.176 | 6.465 | 3.241 | 1.309-8.023 | 0.011 |
| eGFR≧90 | Ref | 72.879 | Ref | Ref | 0.000 |
| 60≦eGFR<90 | 0.320 | 5.166 | 1.377 | 1.045-1.813 | 0.023 |
| eGFR<60 | 1.237 | 64.779 | 3.444 | 2.549-4.654 | 0.000 |
| lipid levels |  |  |  |  |  |
| LDL-C≧3.0mmol/L | -0.173 | 1.869 | 0.841 | 0.656-1.078 | 0.172 |
| HDL-C<1.0 mmol/L | 0.252 | 3.851 | 1.287 | 1.000-1.656 | 0.050 |
| TG≧1.70 mmol/L | 0.386 | 1.855 | 1.471 | 0.844-2.564 | 0.173 |
| Lpa>300 | 0.115 | 0.901 | 1.122 | 0.885-1.423 | 0.343 |
| Results of coronary angiography | | | | | |
| LM lesion | 0.820 | 19.024 | 2.270 | 1.570-3.280 | 0.000 |
| No-reflow phenomenon | 0.656 | 7.617 | 1.926 | 1.209-3.069 | 0.006 |
| Triple-vessel lesions | 0.584 | 23.801 | 1.792 | 1.418-2.266 | 0.000 |
| Complete revascularization | -0.531 | 17.432 | 0.588 | 0.458-0.755 | 0.000 |
| Culprit vessel |  |  |  |  |  |
| LCX | Ref | 37.061 | Ref | Ref | 0.000 |
| RCA | 0.238 | 1.463 | 1.269 | 0.862-1.868 | 0.227 |
| LAD | 0.076 | 0.150 | 1.079 | 0.733-1.589 | 0.699 |
| LM | 1.190 | 12.431 | 3.288 | 1.697-6.373 | 0.000 |
| SVG and others | 2.138 | 23.120 | 8.480 | 3.548-20.268 | 0.000 |
| Degree of vascular stenosis |  |  |  |  |  |
| coronary artery≦70%  LM≦50% | Ref | 2.971 | Ref | Ref | 0.396 |
| 70%<coronary artery≦80%,  50%<LM≦50% | -1.080 | 1.666 | 0.340 | 0.666-1.751 | 0.197 |
| 80%<coronary artery≦90%,  60%<LM≦80% | -0.855 | 1.344 | 0.425 | 0.100-1.805 | 0.246 |
| 90%<coronary artery  80%<LM | -0.617 | 0.756 | 0.539 | 0.134-2.169 | 0.385 |
| Bifurcation lesion | 0.124 | 0.006 | 0.999 | 0.776-1.263 | 0.937 |
| The lesion type |  |  |  |  |  |
| Denove lesion | Ref | 5.821 | Ref | Ref | 0.054 |
| In-stent restenosis | 0.185 | 0.134 | 1.203 | 0.447-3.242 | 0.715 |
| Stent thrombosis | 0.601 | 5.734 | 1.823 | 1.115-2981 | 0.017 |
| Ticagrelor[%(n)] | -0.036 | 0.025 | 0.964 | 0.162-1.518 | 0.875 |

B, correlation coefficient; BP, blood pressure; IABP, Intra-aortic balloon pump; EF, ejection fraction; DTB time, door to balloon time; CKD, chronic kidney disease; Crea, creatinine; eGFR, estimated glomerular filtration rate; PCI, percutaneous coronary intervention; CABG, coronary artery bypass grafting; HDL-C, high-density lipoprotein cholesterol; LDL-C, low-density lipoprotein cholesterol; TG, triglyceride; LPA, lipse activator; hs-CRP, high sensitive C-reactive protein; LM, Left main lesion; LCX, left circumflex coronary artery; RCA, right coronary artery; LAD, left anterior descending branch; SVG, saphenous vein graft; MACE, major adverse cardiovascular events

Appendix table 3 the processes and methods of modeling

| Model | Best.cut.X | Best.cut.sens | Best.cut.spec | N.survivor | N.censored | Cum.incidence | Surv.prob | AUC |
| --- | --- | --- | --- | --- | --- | --- | --- | --- |
| Full | 0.210 | 0.762 | 0.781 | 2864 | 8 | 0.014 | 0.9856 | 0.814 |
| Full | 0.217 | 0.68 | 0.782 | 2790 | 52 | 0.025 | 0.9752 | 0.785 |
| Full | 0.238 | 0.635 | 0.78 | 2005 | 809 | 0.036 | 0.9639 | 0.758 |
| Full | 0.372 | 0.645 | 0.764 | 1350 | 1433 | 0.055 | 0.9454 | 0.758 |
| Full | 0.372 | 0.602 | 0.772 | 1084 | 1671 | 0.077 | 0.9231 | 0.738 |
| Full | 0.740 | 0.723 | 0.597 | 1015 | 1712 | 0.101 | 0.8988 | 0.72 |
| Full | 0.372 | 0.528 | 0.778 | 819 | 1880 | 0.128 | 0.8719 | 0.713 |
| Stepwise | 0.584 | 0.738 | 0.809 | 2864 | 8 | 0.014 | 0.9856 | 0.812 |
| Stepwise | 0.760 | 0.722 | 0.754 | 2790 | 52 | 0.025 | 0.9752 | 0.788 |
| Stepwise | 0.779 | 0.665 | 0.75 | 2005 | 809 | 0.036 | 0.9639 | 0.758 |
| Stepwise | 0.713 | 0.605 | 0.77 | 1350 | 1433 | 0.055 | 0.9454 | 0.74 |
| Stepwise | 0.714 | 0.574 | 0.774 | 1084 | 1671 | 0.077 | 0.9231 | 0.72 |
| Stepwise | 1.161 | 0.695 | 0.6 | 1015 | 1712 | 0.101 | 0.8988 | 0.705 |
| Stepwise | 0.714 | 0.511 | 0.78 | 819 | 1880 | 0.128 | 0.8719 | 0.701 |

AUC, area under curve; Cum.incidence, cumulative incidence; Best.cut.sens, best cutoff sensitive, Best.cut.spec, best cutoff specific; N, number

| **Appendix Figure1 Flow chart of study enrollment.** |
| --- |
| **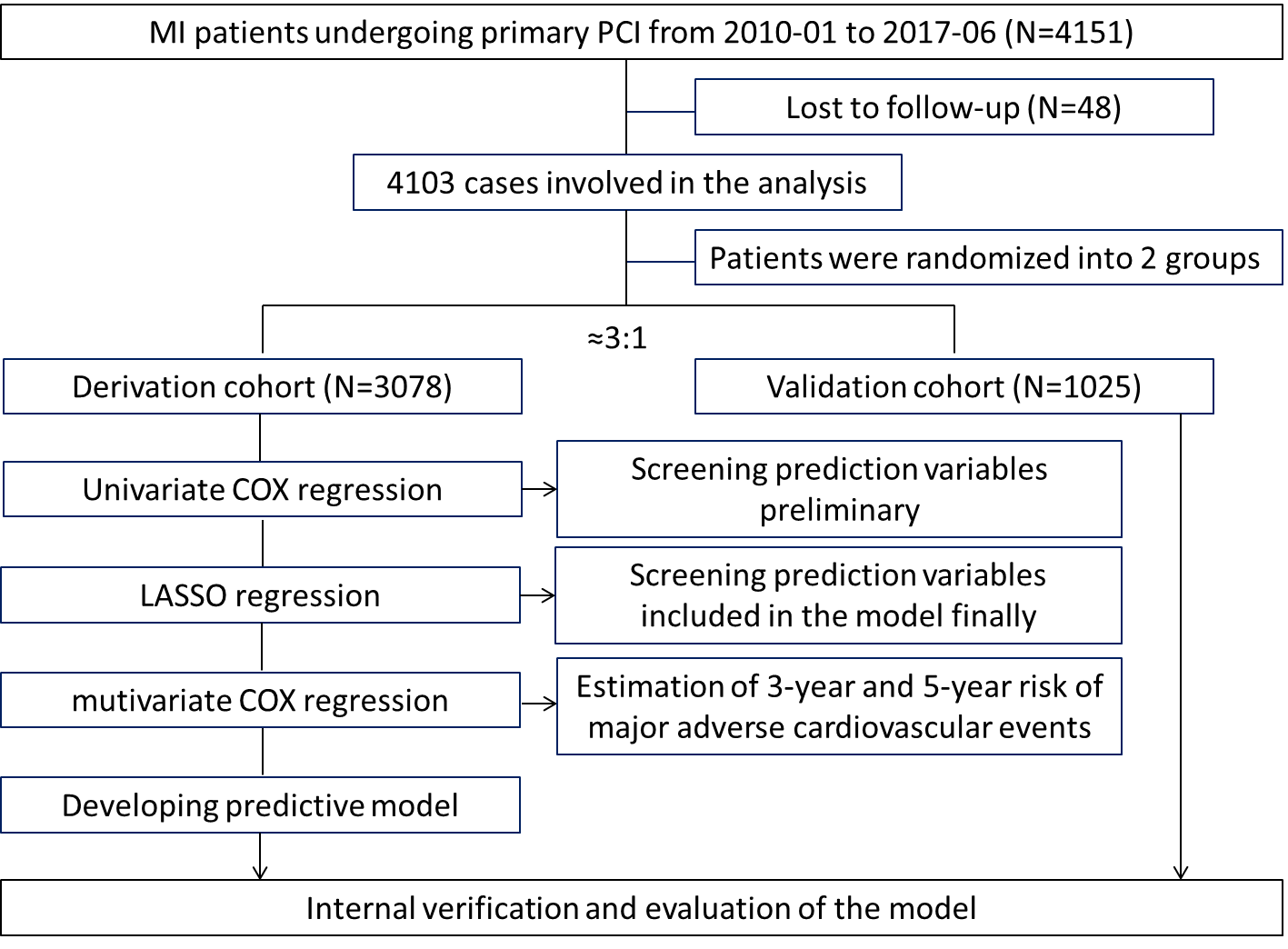**  PCI, percutaneous coronary intervention |
| **Appendix Figure 2 LASSO regression** |
| **A** |
| 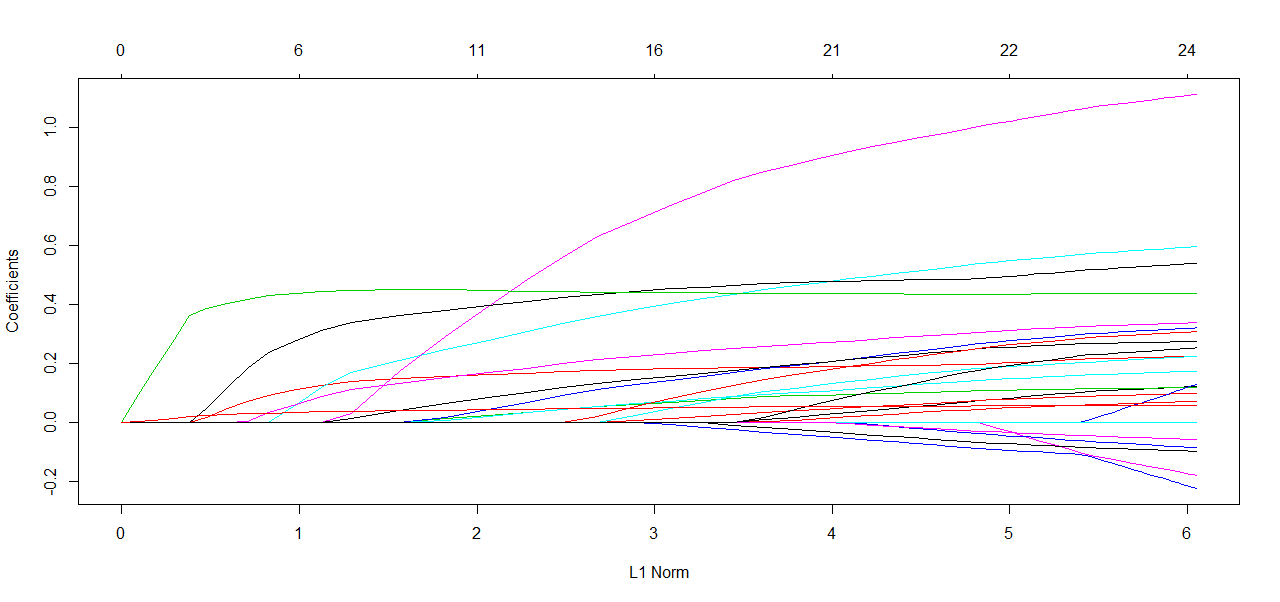  The procedure of selecting independent variables in Cox regression analysis by LASSO method |
| B |
| 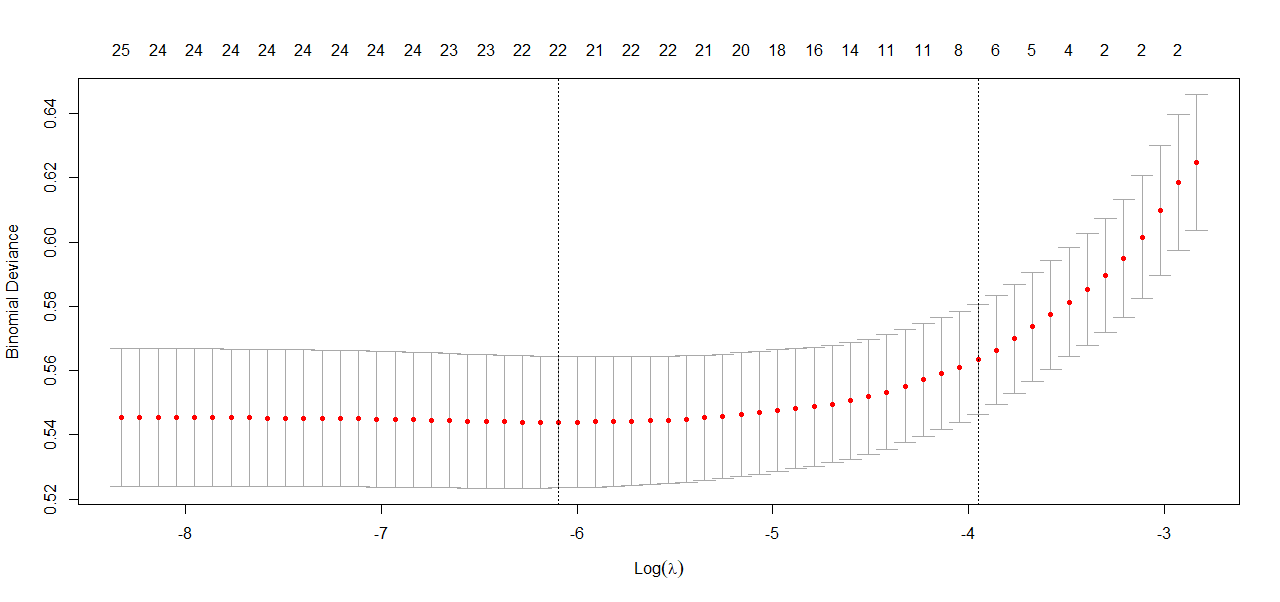  Cross validation process of filtering independent variables by LASSO regression method |

| Appendix figure 3 Survival ROC curve of 3 -year and 5-year cumulative MACE incidence predictive risk in the derivation cohort | |
| --- | --- |
| A | B |
| 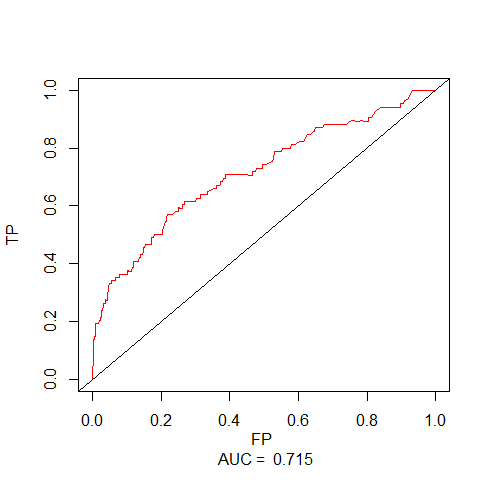 | 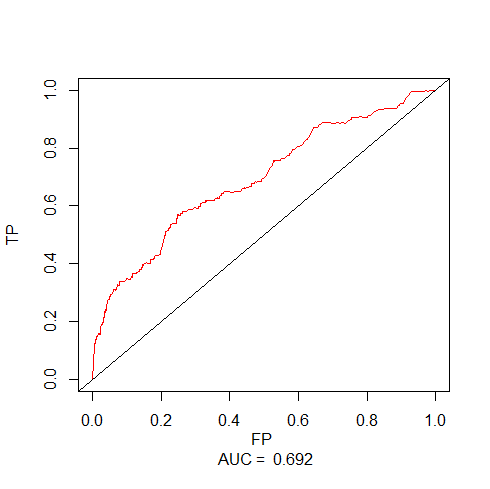 |
| 1-year cutoff point=15.29396 | 2-year cutoff point=15.08561 |
| C | D |
| 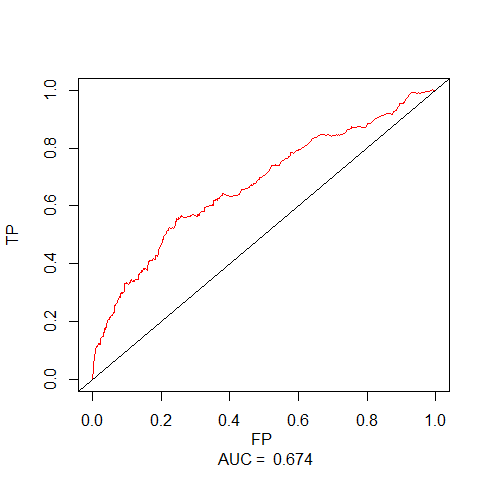 | 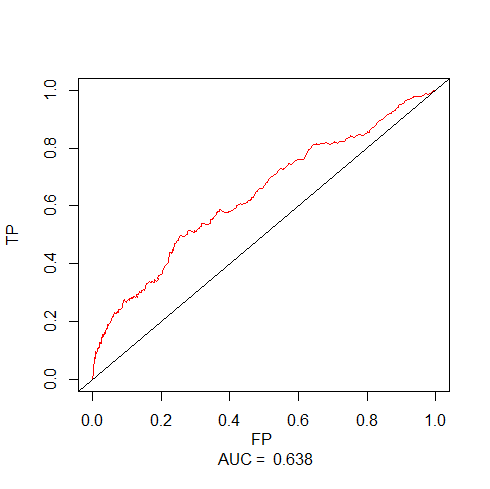 |
| 3-year cutoff point=15.04044 | 5-year cutoff point=14.87151 |
| survival ROC curve  Survival receiver operating characteristic curves for evaluating the performance of the new risk predicted model of 1-year (A, AUC=0.715, cutoff point=15.29396)，2-year (A, AUC=0.692, cutoff point=15.08561)，3-year (A, AUC=0.674, cutoff point=15.04044) and 5-year (B, AUC=0.638, cutoff point=14.87151) in the derivation cohort. The areas under ROC curve are shown for the graph. AUC, area under the curve; ROC, survival receiver operating characteristic; TP, true positive; FP, false positive. | |

Figure 4 Calibration graph in the separate cohort


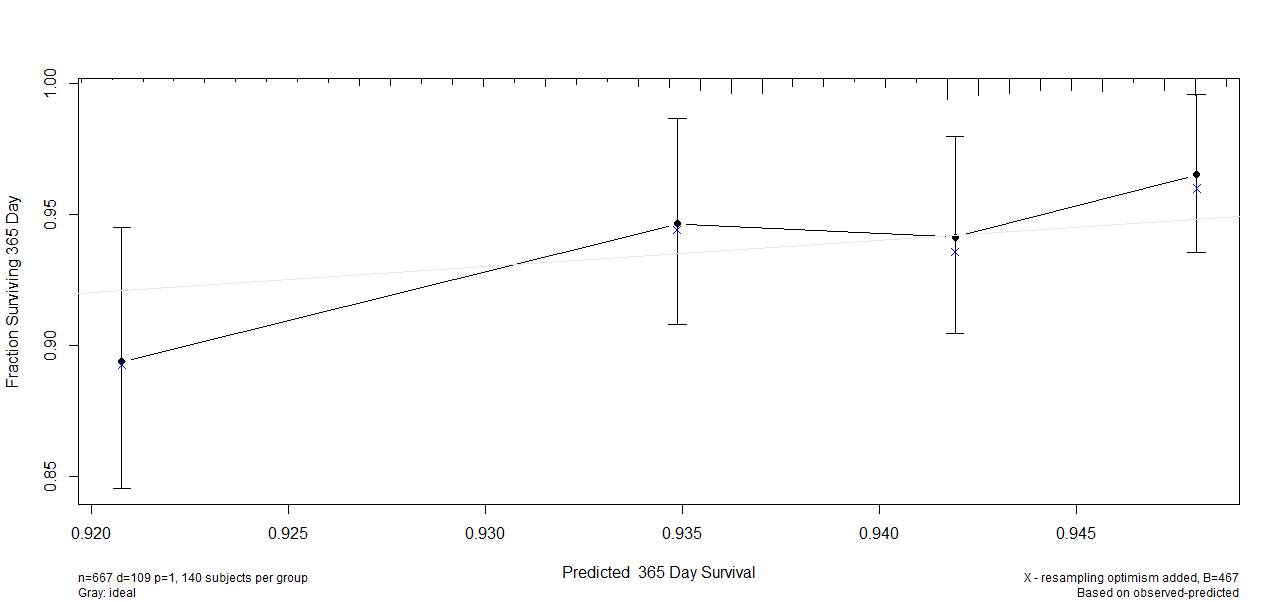


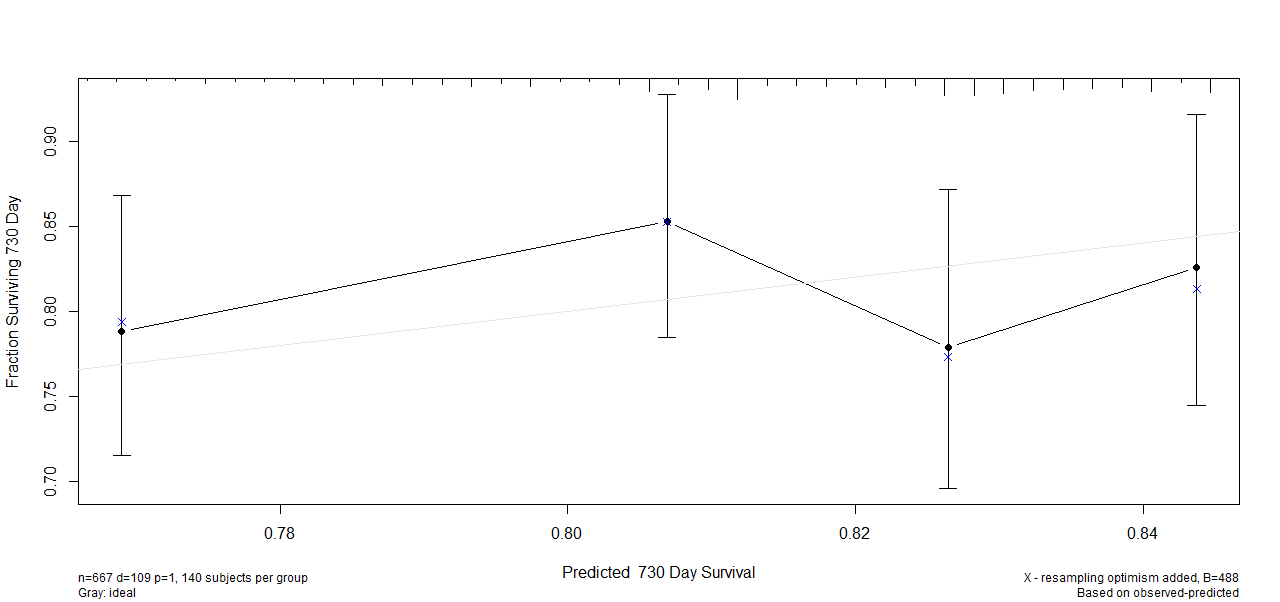

Supplement: Supplementary file 1 [file Table_1.DOCX]
